# Disrupted cognitive and affective empathy network interactions in autistic children viewing social animation

**DOI:** 10.1093/scan/nsae028

**Published:** 2024-04-10

**Authors:** Xinrong Guo, Chuanyong Xu, Jierong Chen, Zhiliu Wu, Shumeng Hou, Zhen Wei

**Affiliations:** Department of Psychology, School of Public Health, Southern Medical University, Guangzhou 510515, China; Department of Child Psychiatry and Rehabilitation, Shenzhen Maternity and Child Healthcare Hospital, Shenzhen 518040, China; School of Psychology, Shenzhen University, Shenzhen 518060, China; Department of Child Psychiatry and Rehabilitation, Shenzhen Maternity and Child Healthcare Hospital, Shenzhen 518040, China; Department of Child Psychiatry and Rehabilitation, Shenzhen Maternity and Child Healthcare Hospital, Shenzhen 518040, China; Department of Humanity and Social Science, Harbin Institute of Technology, Shenzhen 518055, China; Department of Psychology, School of Public Health, Southern Medical University, Guangzhou 510515, China; Department of Child Psychiatry and Rehabilitation, Shenzhen Maternity and Child Healthcare Hospital, Shenzhen 518040, China

**Keywords:** autism spectrum conditions, cognitive empathy, affective empathy, interaction, functional connectivity

## Abstract

Empathy can be divided into two core components, cognitive empathy (CE) and affective empathy (AE), mediated by distinct neural networks. Deficient empathy is a central feature of autism spectrum conditions (ASCs), but it is unclear if this deficit results from disruption solely within empathy networks or from disrupted functional integration between CE and AE networks. To address this issue, we measured functional connectivity (FC) patterns both within and between empathy networks in autistic children (4–8 years, *n* = 31) and matched typically developing (TD) children (*n* = 26) using near-infrared spectroscopy during the presentation of an animated story evoking CE and AE. Empathy and social communication ability were also assessed using the Empathy Quotient/Systemizing Quotient (EQ/SQ) and Social Responsiveness Scale, respectively. The results showed that the FC in the AE network of autistic children did not differ from the TD group across conditions; however, the ASC group showed weaker FC in the CE network under the CE condition and weaker FC between networks when processing AE information, the latter of which was negatively correlated with EQ scores in ASC. The empathy defect in ASC may involve abnormal integration of CE and AE network activities under AE conditions.

## Introduction

Autism spectrum conditions (ASC; Monk *et al*., 2022) are characterized by social cognition and communication impairments, repetitive and stereotyped behaviors, and restricted interests. Autistic individuals have difficulty forming social relationships and communicating with others, further impairing social development and restricting activities of daily life. Atypical empathic behavior in early childhood is a core social impairment in autism (Baron-Cohen, 2010). Indeed, Gillberg (1992) described ASC as a ‘disorder of empathy’. However, the underlying neural mechanisms of these empathy impairments are only beginning to be revealed.

Empathy is a multidimensional construct involving the perception and affective experience of others’ emotional states (affective empathy, AE) as well as the recognition and understanding of others’ mental states (cognitive empathy, CE) (Decety and Moriguchi, 2007; Cuff *et al*., 2016). These two components of empathy appear to be mediated by distinct neural networks (Kanske *et al*., 2016), as CE is strongly associated with activity in the bilateral ventral temporoparietal junction (vTPJ), precuneus, dorsomedial prefrontal cortex (dmPFC), posterior cingulate cortex (PCC) and fusiform gyrus (Greimel *et al*., 2010; Hoffmann *et al*., 2016), while AE is associated with activity in the bilateral ventral medial prefrontal cortex (vmPFC), anterior insula (AI), supramarginal gyrus, dorsal temporoparietal junction, secondary sensory cortex and anterior cingulate cortex (ACC) (Zaki *et al*., 2016; Maliske and Kanske, 2022).

Recent evidence suggests that CE and AE networks are not completely independent but rather interact to process social information, especially when the experimental stimuli are more closely analogous to real-life social interactions (Maliske *et al*., 2023). Evolutionary evidence also suggests that the CE and AE networks must be jointly activated for the accurate understanding of others’ emotions (empathic accuracy) (Zaki *et al*., 2009). Further, Kanske *et al*. (2016) reported that CE can be inhibited by AE in highly emotional situations, indicating that the interactions between AE and CE networks can be both cooperative and competitive. Consistent with important interactions between CE and AE networks, Lamm and colleagues (2011) reported that the CE network is co-activated by visual AE paradigms, suggesting that eliciting an empathic response to abstract visual information about the other’s affective state requires additional cognitive inferences (Lamm *et al*., 2011). These studies suggest that extensive interactions, both cooperative and competitive between CE and AE networks, are essential for social function.

However, in most previous studies of empathy in ASC, CE and AE were investigated as isolated processes (Stietz *et al*., 2019). Impaired CE is a consistent finding in ASC (Greimel *et al*., 2010; Ilzarbe *et al*., 2020; Shi *et al*., 2020; Fatima and Babu, 2023), while studies of AE have yielded inconsistent results (Fatima and Babu, 2023). For instance, Fan and colleagues (2014), reported reduced responses in the anterior mid-cingulate, AI and mPFC of ASC participants compared to typically developing (TD) participants when viewing body parts being injured (AE contents). While others have found no significant differences in brain activation patterns (Hadjikhani *et al*., 2014), network connectivity (Hoffmann *et al*., 2016) or skin conductance (Trimmer *et al*., 2017) between ASC and TD groups. Furthermore, a large-scale study of the resting-state connectome in ASC-reported reduced functional connectivity (FC) among regions of the CE network but no measurable differences in the AE network (Hoffmann *et al*., 2016). In contrast, a recent meta-analysis concluded that FC values within both CE and AE networks were significantly weaker in the ASC group than the control group (Fatima and Babu, 2023). Although interactions between CE and AE networks have been studied extensively in TD individuals, it is unclear if the integration between CE and AE during particular empathy tasks is disrupted in ASC. In light of the inconsistencies in the results of empathy network connections among autistic individuals, additional comprehensive studies on intra network connections and inter network interactions are needed to elucidate the detailed characteristics and neurophysiological mechanisms of empathy in ASC. Such disruption may explain the disparities across studies and identify novel pathomechanisms for ASC.

To examine the interactions between CE and AE networks, we compared the neural activity and FC patterns of autistic children to those of matched TD children while viewing an animated movie containing events engaging CE and AE. This animated stimulus was chosen to minimize cognitive load while still providing sufficient stimulation for improved compliance. Moreover, we used functional near-infrared spectroscopy (fNIRS) to measure neural network activity (Vanderwal *et al*., 2015) as this optical imaging technique for detecting cortical hemodynamic responses or blood-oxygenation level-dependent (BOLD) responses associated with regional neuronal activation (Raichle and Mintun, 2006) is less sensitive to movement artifacts and does not require children to lie within a confined space. These attributes are particularly advantageous as autistic children have difficulty maintaining the complete immobility required for functional magnetic resonance imaging (fMRI). Moreover, several studies have successfully measured the processing of social information by autistic children using fNIRS devices, including social perception, face processing and inferring the mental states of others (Iwanaga *et al*., 2013; Zhang and Roeyers, 2019).

Based on previous work, we derived three hypotheses regarding empathy network changes in autistic children. First, we expected to replicate previous findings of reduced FC within the CE network. Moreover, we speculated that interactions between CE and AE networks would be influenced by different empathy stimuli. Finally, while previous evidence is equivocal, we hypothesized that inter-network FC would be disrupted in ASC and associated with characteristics of empathy and social communication.

## Methods

### Participants

A total of 35 autistic children and 27 TD children were recruited in this study. Four autistic participants were removed for including excessive noise or saturation artifacts in NIRS data, and one TD participant was excluded due to meeting the exclusion criteria for a diagnosis of global developmental delay. This resulted in a final sample of 31 autistic children (4.01–7.54 years, 4 females) and 26 TD children matched for age and sex ratio (4.21–7.93 years, 8 females). Relevant demographic and clinical variables are summarized in Table 1. From 2022 to 2023, autistic children were recruited from Shenzhen Maternity and Child Healthcare Hospital, while TD children were recruited from the local community through posters and the Internet.

**Table 1.** Demographic and clinical characteristics of ASC and TD groups

	Group			
	ASC (*n* = 31)	TD (*n* = 26)	*t/χ* ^2^	*P*-values
Age (years)	5.52 (1.1)	6.04 (1.2)	−1.734	0.089
Sex (no. of male/female)	27/4	18/8	2.716	0.099
DQ (no. of ASC/TD = 17/16)	57.94 (12.9)	90.81 (8.7)	−8.527	<0.001^**^
FSIQ (no. of ASC/TD = 10/9)	79.90 (17.5)	116.44 (12.3)	−5.199	<0.001^**^
EQ	14.20 (6.1)	28.46 (7.6)	−7.771	<0.001^**^
CE	3.33 (3.0)	12.23 (3.7)	−9.984	<0.001^**^
AE	3.10 (2.3)	7.42 (2.8)	−6.346	<0.001^**^
SRS	80.96 (25.2)	34.04 (17.0)	7.720	<0.001^**^
CARS	34.72 (3.9)	–	–	–

Notes. All values are expressed as mean (SD) except for sex (*n*). DQ = Developmental Quotient; FSIQ = Full-scale Intelligence Quotient; EQ = Empathy Quotient; SRS = Social Responsiveness Scale; CARS = Childhood Autism Rating Scale. The TD group was significantly older than the autism group (*t *= 2.64, *P *= 0.005; *t *= 0.80, *P *= 0.788) when age equivalence was tested using a two one-sided *t*-test (TOST; Lakens *et al*., 2018). Age and sex were included as covariates in subsequent analyses.

** *P *< 0.01 by independent samples Student’s *t*-test or *χ*^2^ test.

All children in the ASC group were diagnosed by two experienced hospital psychiatrists based on DSM-5 criteria (American Psychiatric Association, 2013), and further assessed by the Autism Diagnostic Observation Schedule, Second Edition (ADOS-2; Lord *et al*., 2000) and Childhood Autism Rating Scale (CARS; Schopler *et al*., 2010). All participants were right-handed and met the NIRS inspection requirements. Participants were free from the following exclusion criteria: (i) history of neurological disease or major physical illness; (ii) other mental disorders (e.g. schizophrenia, depression or phobias, tic disorders, etc.); (iii) history of alcohol and drug abuse or dependence. Additional exclusion criteria for control participants were any current or past psychiatric disorder and a family history of autism. Written informed consent was obtained from all parents or guardians prior to psychometric and fNIRS examinations, and all study protocols were approved by the research ethics committee of Shenzhen Maternity and Child Healthcare Hospital.

### Materials and measures

#### Empathy and social ability measures

The Chinese versions of the Children’s Empathy Quotient and Systemizing Quotient (EQ/SQ-C; Wang *et al*., 2022) and the Social Responsiveness Scale (SRS; Constantino, 2002) were completed by parents to evaluate their child’s empathy and social communication ability. The EQ/SQ-C was developed by Baron-Cohen and colleagues (Baron-Cohen *et al*., 2003; Baron-Cohen and Wheelwright, 2004), revised for children by Auyeung *et al*. (2009), and finally adapted to Chinese according to the characteristics of children in the Chinese mainland (Wang *et al*., 2022). The empathy subscale includes 23 items assessing three factors: CE, AE and social skills. Items are scored from 0 to 2, with higher scores indicating greater empathic capacity. The SRS total score can serve as an index of social-deficit severity in the autism spectrum as it has been shown to be strongly associated with the results of the Autism Diagnostic Interview-Revised (ADI-R; Constantino *et al*., 2003). A higher SRS score is indicative of more serious social interaction impairments. As recommended, we report the raw SRS score instead of the converted T-score for comparability with other studies (Zhou *et al*., 2017).

#### Cognitive measurements

Developmental state was evaluated using Chinese versions of the Gesell Developmental Scale (GDS-C; Zhang *et al*., 1994) for children ≤6 years of age and Wechsler Intelligence Scale, Fourth version (WISC-IV; Zhang, 2009) for children >6 years of age. Both versions have demonstrated strong reliability and validity for children in the Chinese mainland. The GDS-C measures the developmental quotient (DQ) of children through five domains, adaptive behavior, gross motor, fine motor, language behavior, and personal-social behavior, while the WISC-IV measures the full-scale intelligence quotient (FSIQ) based on index scores for verbal comprehension, perceptual reasoning, working memory and processing speed. As the scores of the two scales could not be converted into each other, we finally analyzed the effect of intelligence using a dichotomous method. Children in the ASC group with DQ score ≤75 or FSIQ <70 were classified as ‘ASC with intellectual disability (ID)’ in further analysis to control for the effects of intelligence.

#### Experiment stimuli

All children viewed a silent version of *Partly Cloudy*, a 5-min animated movie from Pixar (https://www.pixar.com/partly-cloudy#partly-cloudy-1). A previous study using reverse correlation analyses extracted seven CE events [68 s total, *M* (s.d.) length *9.7* (4.2) s] and twelve AE events [86 s total, *M* (s.d.) length *7.2* (4.7) s] from the movie that can evoke activity in brain regions associated with empathy (Richardson *et al*., 2018). CE events depict changes in characters’ beliefs, desires, or complex emotions (e.g. a baby stops crying when given a helmet) while AE events depict characters’ feelings and emotions (e.g. a stork named Peck tosses a porcupine baby and expresses pain; a cloud named Gus begins to cry heavily, thereby making it rain). We measured oxyhemoglobin changes in cortical regions known to be associated with CE and AE in typically developed children (shown in Supplementary Figure S1) using a portable fNIRS system.

### fNIRS data acquisition and analysis

#### fNIRS data acquisition

**Fig. 1.**
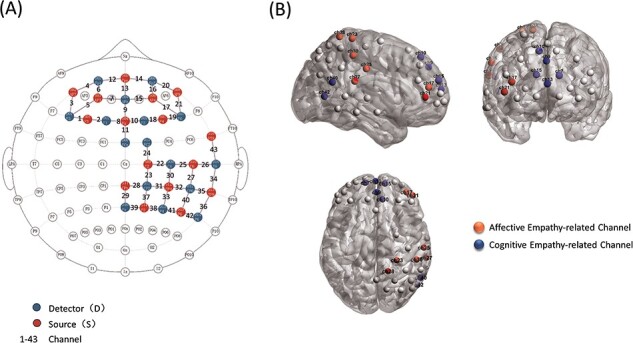
Locations of fNIRS channels. (A) Positions of the NIRS sources, detectors and channels. (B) Channel projections on Colin27 templates. Cognitive empathy-related channels were distributed mainly over the dorsolateral superior frontal gyrus, medial superior frontal gyrus and middle temporal gyrus, while affective empathy-related channels were distributed mainly over the middle frontal gyrus, postcentral gyrus, supramarginal gyrus, superior parietal gyrus and inferior parietal cortex (supramarginal and angular gyri).

A continuous-wave NIRSport system was used to measure relative concentrations of oxyhemoglobin (HbO) and deoxygenated hemoglobin (HbR) (NIRSport, NIRx Medical Technologies, Glen Head, NY, USA) at a sampling rate of 3.47 Hz measuring at two wavelengths (760 nm and 850 nm). The NIRSport system consists of 16 near-infrared light sources and 16 detectors. Light sources and detectors were placed in a textile EEG cap of appropriate size for each participant (EASYCAP, Herrsching, Germany), forming an array of 43 channels (Figure 1A). Montreal Neurological Institute (MNI) coordinates of each channel and the corresponding brain regions from the automated anatomical labeling template (AAL2) were exported from the MATLAB toolbox fOLD (fNIRS Optodes’ Location Decider). Based on previous brain network research using the same experimental paradigm (Richardson *et al*., 2018), we then entered the MNI coordinates of each channel in Neurosynth (http://neurosynth.org/; search term: ‘tom,’ forward inference from 80 studies, referring to theory of mind, which corresponds to CE; ‘pain,’ forward inference from 516 studies, corresponding to AE) for functional localization. Channel 8 was removed due to signal weakness and Channel 13 was assigned to the CE network as it was more sensitive to CE network activation despite covering regions belonging to both empathy networks. Finally, we selected seven channels related to CE brain regions (mainly distributed in the dorsolateral superior frontal gyrus, medial superior frontal gyrus and middle temporal gyrus) and seven channels related to AE brain regions (mainly distributed in the middle frontal gyrus, postcentral gyrus, supramarginal gyrus, superior parietal gyrus and angular gyrus). A three-dimensional location diagram is presented in Figure 1B, while MNI coordinates, corresponding brain regions and posterior probability with the term ‘empathy’ in Neurosynth are listed in Supplementary Table S1. The results of posterior probability show that for each channel, close to or more than half of the studies are suggestive of a relationship between channel activity and empathy, further suggesting that the channels we have selected are part of the empathy network.

#### Signal preprocessing

The fNIRS signals were preprocessed in eight steps according to recent guidelines (Di Lorenzo *et al*., 2019) using the Homer2 package in MATLAB (R2013b). First, raw signal intensities were converted to optical densities (OD values) using *hmR_Intensity2OD*, and channels with abnormally high or low values were excluded using the *enPruneChannels* command (dRange: 1e-02 to 1e + 07; SNRthresh: 2; SDrange: 0.0–45.0). Channels were then checked for motion artifacts using the *hmrMotionArtifactByChannel* function (tMotion: 3.0; tMask: 1.0; STDEVthresh 13.0; AMPthresh: 0.40) and the combination of Spline (*hmrMotionCorrectSpline*, p: 0.99), and Wavelet (*hmrMotionCorrectWavelet*, iqr: 0.80) functions were followed for motion artifacts processing. The *hmrMotionArtifactByChannel* function was used again to check for remaining motion artifacts. Next, signals were band-pass filtered using *hmrBandpassFilt* (hpf: 0.010, lpf: 0.10) to reduce physiological noise. Finally, OD values were converted to blood oxygen concentrations using *hmrOD2Conc* [ppf (5.5 4.9)]. The differential path length factor (DPF) was measured and corrected for average age and wavelengths (Duncan *et al*., 1996).

#### Functional connectivity analysis

The preprocessed signals were then analyzed to reconstruct network activity patterns using a custom script written in MATLAB. FC was analyzed under the general conditions (154 s, 565 scans, including all empathic events), CE condition (68 s, 245 scans) and AE condition (86 s, 320 scans). In each condition, a 14 × 14 matrix was obtained by correlation analysis of signals from 14 channels and normalized by Fisher *z*-transform. To reduce spurious associations, each correlation in the matrix was compared to 0 (null hypothesis) by one-sample *t*-test. Inter-channel correlation analyses and *P*-values from one-sample *t*-tests are shown in Supplementary Figure S2. There were three negative connecting edges in the matrix, all of which were inter-network connections and non-significant, which were eliminated under three conditions. After removing non-significant edges, the remaining connections were all positive and divided into three types: within CE network, within AE network and across networks. Multivariate ANOVA was used to assess group differences in average connectivity strength within each network under general, CE and AE conditions. Statistical results were corrected for multiple comparisons using the Benjamini/Hochberg false discovery rate (BH-FDR) procedure. We then performed edge-by-edge group comparisons of the correlation matrices using a network-based statistic (NBS) method (connection *t* > 2.5, cluster *P* < 0.05, 5000 permutations).

#### Correlations between behavioral and fNIRS results

To examine associations of ASC characteristics with empathy network properties, correlations were calculated between connection strengths and total EQ score, AE and CE subscale scores, total SRS score and CARS score. Age and sex were included as covariates in all analyses as these factors are well known to influence empathy impairments in autistic individuals (Song *et al*., 2019).

## Results

### Comprehensive empathy and social communication scored lower in autistic children

Compared to TD children, autistic children needed specific support in general cognitive ability as evidenced by lower DQ (*t* = − 8.527, *P* = 1.2 × 10^−9^) and FSIQ (*t* = − 5.199, *p* = 7.2 × 10^−5^), general empathy as indicated by a lower EQ (*t* = − 7.771, *P* = 2.3 × 10^−10^) as well as poor CE (*t* = − 9.984, *p* = 7.2 × 10^−14^) and AE (*t* = − 6.346, *P* = 4.7 × 10^−8^) subscale scores, and social response ability as indicated by lower SRS score (*t* = 7.720, *P* = 5.8 × 10^−10^) (Table 1). Further, EQ was negatively correlated with SRS score (*r* = − 0.791, *P* = 1.3 × 10^−11^, controlling for age and sex as covariates). Autistic children without ID scored lower on the CARS compared to autistic children with ID (32.25 ± 2.4 *vs* 36.17 ± 4.0), while no subgroup difference was found in age, EQ total score, AE subscore, CE subscore or SRS score (Supplementary Table S2). These findings suggest that the empathy impairments in ASC are not explained by lower general intelligence.

### Intact AE network in autistic children across conditions

**Fig. 2.**
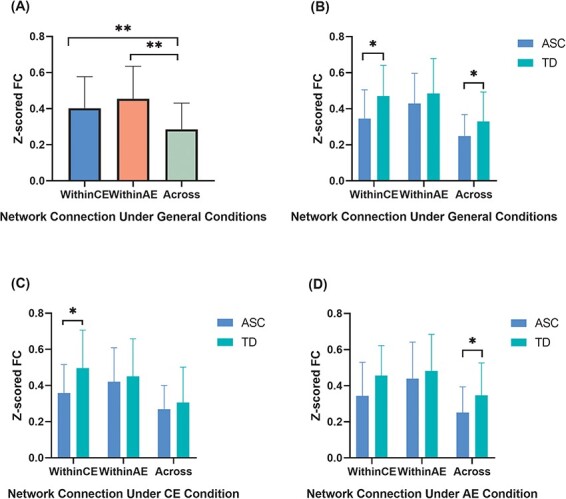
Reduced functional connectivity (FC) strengths both within and between empathy networks among children with ASC. (A) Intra-network connectivity was significantly stronger than inter-network connectivity for the entire cohort under general conditions (presentation of events evoking both CE and AE). (B–D) Intra-network connectivity was also significantly stronger than inter-network connectivity in both ASC and TD groups (B) under general conditions, (C) during presentation of events evoking CE (cognitive empathy condition) and (D) during presentation of events evoking AE (affective empathy condition). Connectivity within the CE network was weaker in children with ASC compared to TD children under general conditions (B) and the CE condition (C) but not the AE condition (D). Connectivity between CE and AE networks was also weaker in children with ASC under (B) general conditions and (D) the AE condition. Error bars represent standard deviation (SD). *P < 0.05, **P < 0.01 with false discovery rate multiple comparison correction.

As shown in Figure 2A, mean connectivity strengths within CE and AE networks were significantly greater than between the CE and AE networks (*F* = 15.188, *P* = 8.8 × 10^−7^ by ANOVA including age and sex as covariates; within-CE *vs* between networks: *P* < 0.001 by post hoc test; within-AE *vs* between networks: *P* = 7.5 × 10^−7^ by post hoc test). Comparisons between groups revealed that the ASC group showed intact AE network connectivity across conditions (under general conditions: *p*-fdr = 0.254; under CE condition: *p*-fdr dr0.254; under CE condition: *p*-fdr = 0.525) (Figure 2B–D). Further edge-by-edge analyses also did not find significantly different connecting edges within the AE network.

### Altered CE intra-network connectivity under the CE condition and inter-network connectivity under the AE condition

Analysis of fNIRS signals during both CE and AE events in the stimulus film (referred to as general conditions) revealed reduced mean connectivity within the CE network (*p*-fdr = 0.0315) and between the CE and AE networks (*p*-fdr = 0.0315) of autistic children (Figure 2B). Further analysis also revealed a significant group effect on connectivity strength within the CE network under the CE condition (analysis of fNIRS signals only during CE events, *p*-fdr = 0.045) and between networks under the AE condition (analysis of fNIRS signals only during AE events, *p*-fdr = 0.045) (Figure 2C and D). In addition, connectivity strength within the CE network was also numerically lower in autistic children under the AE condition (*p*-fdr = 0.0855).

**Fig. 3.**
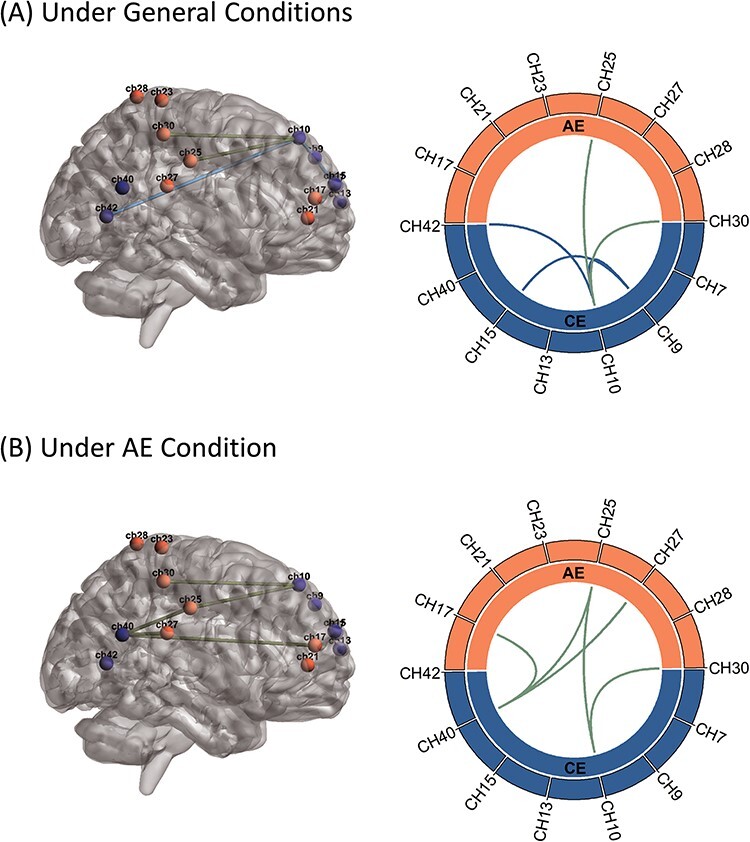
Children with ASC exhibited weaker individual functional connections (edges) between CE and AE networks and within the CE network. (A, B) Group differences in edge-by-edge functional connectivity between region-of-interest (ROI) pairs under (A) general conditions and (B) the affective empathy condition. CH, channel.

Edge-by-edge analysis also revealed that five edges (between channels 10 and 25, 10 and 30, 9 and 10, 9 and 15 and 10 and 42) were significantly weaker in autistic children under general conditions (Figure 3A) and five edges (between channels 10 and 25, 10 and 30, 40 and 17, 40 and 25 and 40 and 27) were weaker under the AE condition (Figure 3B), while no differences in edge strength were found under the CE condition. Consistent with the analysis of network-averaged connectivity, these weaker edges were found mainly between networks under the AE condition and within the CE network under general conditions.

### Correlations between altered FC and autistic characteristics

**Fig. 4.**
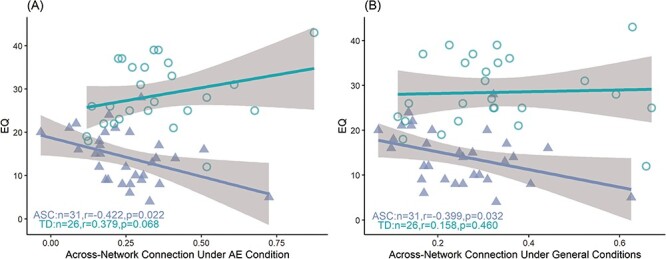
The correlation between inter-network functional connectivity strength of CE and AE networks and empathy shows different trends in ASC and TD groups. (A, B) Scatter plots showing the correlations between inter-network connectivity strength and EQ total score under the AE condition (A) and general conditions (B).

Correlation analysis revealed that the EQ score was negatively associated with inter-network FC strength among autistic children (i.e. lower empathy was associated with stronger FC) under the AE condition (*r* = − 0.422, *P* = 0.022 with sex and age as covariates) and general conditions (*r* = − 0.399, *P* = 0.032 with sex and age as covariates). In contrast, this association was positive in TD children both under the AE condition (*r* = 0.379, *P* = 0.068 with sex and age as covariates) and general conditions (*r* = 0.158, *P* = 0.460 with sex and age as covariates) (Figure 4). No significant correlations were found between mean network FC strength, and AE, CE, SRS and CARS scores in either group (Supplementary Table S3).

## Discussion

In the current study, we measured the connectivity characteristics within and between empathy sub-networks in autistic individuals under two conditions: CE and AE. Our results demonstrated that autistic individuals show lower connectivity within CE networks under the CE condition and between empathy sub-networks under AE condition, the latter of which was related to their empathy ability. These findings may not only help explain discrepancies among previous studies on empathy characteristics in ASC but also provide clues to the underlying neuronal mechanisms and therapeutic targets.

Most previous studies have explored connections within CE network under CE condition and connections within AE network under AE condition (Hadjikhani *et al*., 2014; Ilzarbe *et al*., 2020), and in the present study, we examined the connections within the two networks under each of the two conditions. As with previous research results (Hoffmann *et al*., 2016; Ilzarbe *et al*., 2020), we found weak connections in the CE network of the ASC group under CE condition. When processing AE events, there was no significant difference in the connections within the CE network between the ASC group and TD group. Considering the possible interactions between the two sub-networks of empathy, in addition to examining the network connections for the brain regions consistent with the condition, an examination of the connections within the other empathic network, which is not consistent with the condition, is also warranted (Völlm *et al*., 2006; Maliske *et al*., 2023). Previous studies have yielded inconsistent results regarding responses to AE-inducing stimuli by autistic individuals, with some reporting hyperarousal, others normal arousal and still others hypoarousal during AE processing (Fan *et al*., 2014; Rueda *et al*., 2015; Hoffmann *et al*., 2016; Trimmer *et al*., 2017). Our results are most consistent with the second outcome and the results are consistent across conditions, as autistic children exhibited normal connection strength in response to emotionally salient stimuli. We speculate that not accounting for interactions between CE and AE networks in the experimental design (stimulus paradigm) may account for the inconsistencies reported across previous studies.

To investigate interactions between CE and AE networks, we measured FC at both whole-network and edge-by-edge levels using fNIRS. These analyses revealed significantly weaker connectivity between CE and AE networks during the presentation of stimuli evoking AE among autistic children. In most previous studies, only AE network activity was examined during AE tasks (Bruneau *et al*., 2012; Hadjikhani *et al*., 2014). However, Lamm and colleagues (2011) did report additional recruitment of the CE network during the processing of stimuli evoking AE. The positive coupling of modular networks (‘integration’) is associated with cognitively demanding tasks requiring more effortful and controlled processing (Fornito *et al*., 2012; Shine *et al*., 2016; Maliske *et al*., 2023), and AE tasks can be cognitively demanding due to the additional requirement for cognitive processing, such as the analysis of social cues (Lamm *et al*., 2007). In light of these findings and those of the current study, it is reasonable to speculate that the processing of AE stimuli requires integration of CE and AE network activities and that this capacity is diminished in autistic children. Indeed, reduced functional integration in ASC has been observed for the face perception (Nickl-Jockschat *et al*., 2015) and emotion processing networks (Rudie *et al*., 2012), possibly due to impaired FC among amygdala, secondary visual areas, occipital regions, inferior frontal cortex and limbic regions among others. However, neither study examined network integration during empathy tasks.

Previous studies of TD children have indicated that neural networks can be positively coupled (for integration or cooperative activity, measuring ‘global efficiency’) or negatively coupled (resulting in functional segregation, measuring ‘local efficiency’) (Trujillo *et al*., 2022; Maliske *et al*., 2023). As is shown in Figure 2A, the connection across networks was weaker than the mean connectivity strength within both cognitive and AE networks, which would confer segregation. In the present study, correlation analysis revealed a positive association between psychometric empathy score and functional integration strength between empathy sub-networks in the TD group (i.e. stronger inter-network connectivity was associated with greater empathy), but surprisingly, this association was negative in the ASC group. Thus, while stronger integration between networks appears essential for empathy among TD children, stronger segregation appears essential for empathy in ASC. This may be because autistic individuals utilize a more local-processing-oriented network configuration (i.e. higher local efficiency) rather than the more integrative network organization seen in neurotypicals (i.e. higher global efficiency) (Wadhera and Kakkar, 2021; Trujillo *et al*., 2022), further representing that empathy network with short functional reach might be a compensation mechanism for processing empathy information in ASC, thus showing that weaker integration between empathic sub-networks is associated with better empathic ability. These results shed new light on the complex interplay between cognitive and AE processing in autism.

The empathy stimuli that are closer to real-life social interactions better reflect the empathy ability of autistic individuals in their daily lives. Dynamic or video stimuli are starting to be used (Hadjikhani *et al*., 2014). In the animation stimuli of this study, the ‘empathy events’ were identified based on the article by Richardson *et al*. (2018), in which they confirmed that these events were able to activate cognitive and AE-related brain regions, respectively, in 3- to 12-year-old TD children. However, video materials are always difficult to finely manipulate and control the content. The current research can only explore whether autistic children exhibit different characteristics from TD children in CE or AE at the level of brain networks, but have not provide a more detailed distinction between the various steps involved in CE and AE, like recognizing, interpreting or responding. Future research can refine empathy events in video experiments or combine brain activity with behavioral experiments or eye tracking for further analysis.

This study has several important limitations. First, EQ was determined by a parent-reported scale, and neurotypical individuals often fail to accurately predict the emotional and mental states of autistic individuals (Sheppard *et al*., 2016). Therefore, more objective behavioral measures are required in future studies (Fletcher-Watson and Bird, 2020). Second, the channels we choose through the terms ‘tom’ and ‘pain’ are part of the CE and AE brain networks, but may not fully represent them. In addition, fNIRS cannot detect activation below the neocortex, which includes core brain regions for AE such as the insula and for CE such as the precuneus (Maliske and Kanske, 2022). Therefore, future studies are needed to assess activity and connectivity within entire AE and CE networks using other modalities such as fMRI. Finally, a meta-analytic clustering of neuroimaging data further stratified AE tasks into purely affective and cue-based, and found that only cue-based AE tasks additionally recruited the CE network (Schurz *et al*., 2021). Therefore, future research should also include cue-based AE tasks.

In conclusion, the current study revealed atypical connectivity within and between neural networks for empathy in young autistic children. Specifically, these autistic children exhibited normal FC within the AE network across conditions but significantly reduced FC within the CE network under CE condition as well as aberrant FC between networks that was correlated with low empathy under AE condition. Autistic children in our sample may lack efficient information integration capacity between CE and AE networks when processing AE. These findings may help explain inconsistent results in previous studies on AE in ASC and provide important guidance for future research, in particular addressing how these altered inter-network interactions lead to poor empathy and whether targeting the CE–AE network pathway is an effective therapeutic intervention.

## Supplementary Material

nsae028_Supp

## Data Availability

All data generated for this study are presented in the figures and tables. Supplementary data are shown in the ‘Supplementary data’ document.
