# Supplementary material for: Disrupted cognitive and affective empathy network interactions in autistic children viewing social animation
Source: Soc Cogn Affect Neurosci. 2024 Apr 10;19(1):nsae028. doi: 10.1093/scan/nsae028 (PMC11071513; doi:10.1093/scan/nsae028)
Supplement: nsae028_Supp [file nsae028_supp.zip › scan-23-229-File007.docx]

**Supplementary Materials for “ Disrupted cognitive and affective empathy network interactions in autistic children viewing social animation ”**

**Supplementary Figure 1**

**
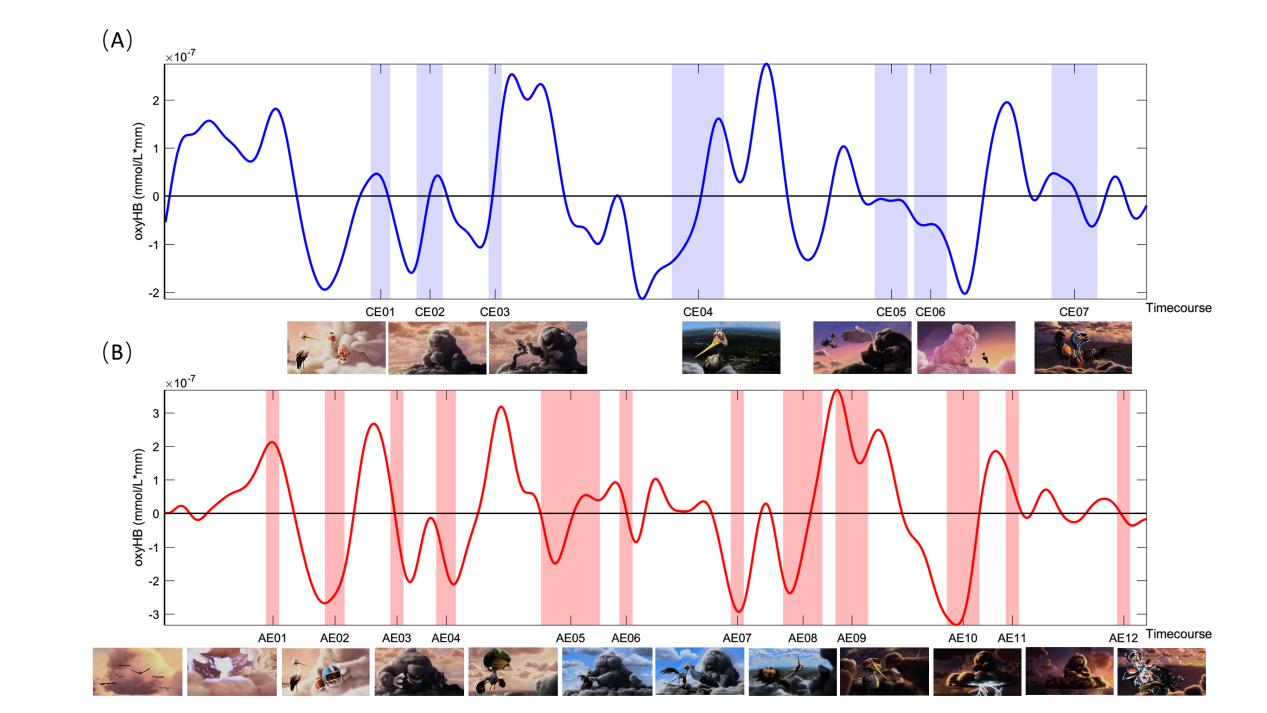
**

**Fig. S1.** The average time course of oxygenated hemoglobin (OxyHb) concentration changes for cognitive empathy (CE) network (A) and affective empathy (AE) network (B), during viewing of ‘*Partly Cloudy*’ in typical developing group (N=26). Shaded blocks show timepoints identified as cognitive empathy (blue) and affective empathy (red) events. The picture below is a screenshot of the event.

**Supplementary Figure 2**


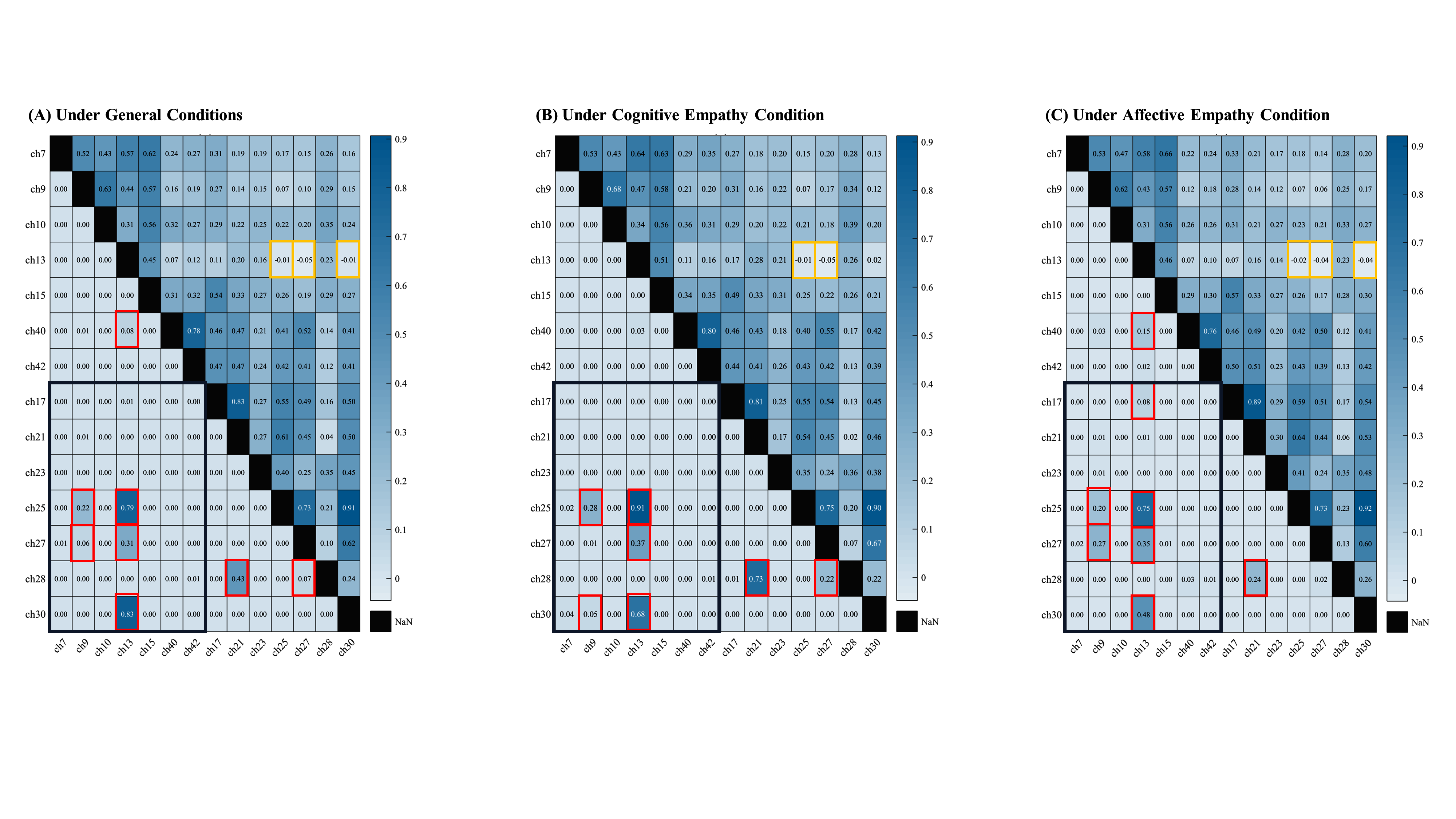


**Fig. S2.** Inter-channel correlation analysis (upper triangles) and p-values from one-sample t-tests against 0 (lower triangles; corrected by BH-FDR) under general condition (A), cognitive empathy condition (B) and affective empathy condition (C). Non-significant connecting edges (*p_*fdr > 0.05) are in the red boxes and negative connecting edges are in the yellow boxes.

After removing non-significant edges, there are a total of 55 remaining connections under general condition (within CE network (19 edges), within AE network (20 edges), and across networks (16 edges)), 56 remaining connections under the CE condition (within CE network (19 edges), within AE network (21 edges), and across networks (16 edges)), and 55 remaining connections under the AE condition (within CE network (20 edges), within AE network (20 edges), and across networks (15 edges)). All the connections are positive.

**Table S1.** MNI coordinate and corresponding brain area in AAL2 of channels.

| Belonged network | Channel | MNI coordinate  [x y z] | Corresponding brain area in AAL2 | Posterior probability with the term “empathy” |
| --- | --- | --- | --- | --- |
| CE network | CH07 | [-12 62 23] | SFGdor.L | 0.74 |
|  | CH09 | [2 50 39] | SFGmed.L | 0.65 |
|  | CH10 | [10 41 50] | SFGmed.R | 0.58 |
|  | CH13 | [1 64 14] | SFGmed.L | 0.66 |
|  | CH15 | [13 61 24] | SFGdor.R | 0.61 |
|  | CH40 | [58 -58 22] | MTG.R | 0.70 |
|  | CH42 | [54 -67 6] | MTG.R | 0.63 |
| AE network | CH17 | [40 50 16] | MFG.R | 0.60 |
|  | CH21 | [48 46 5] | MFG.R | 0.49 |
|  | CH23 | [28 -36 71] | PoCG.R | 0.57 |
|  | CH25 | [62 -20 37] | SMG.R | 0.85 |
|  | CH27 | [65 -33 23] | SMG.R | 0.43 |
|  | CH28 | [17 -50 73] | SPG.R | 0.50 |
|  | CH30 | [53 -35 52] | IPL.R | 0.45 |

*Note*, Posterior probability is a metric in Neurosynth that refers to the probability of studies that report activation at the current location also use the term 'empathy' in their abstracts. CE, cognitive empathy; AE, affective empathy; L, the left hemisphere; R, the right hemisphere; SFGdor, superior frontal gyrus, dorsolateral; SFGmed, superior frontal gyrus, medial; MTG, middle temporal gyrus; MFG, middle frontal gyrus; PoCG, Postcentral gyrus; SMG, supramarginal gyrus; SPG, superior parietal gyrus; IPL, inferior parietal, but supramarginal and angular gyri.

**Table S2.** The comparison of demographic and clinical scores and network connections with significant group difference (ASC vs. TD) in autistic children with and without ID

| condition |  | ASC without ID  (N=12) | ASC with ID  (N=18) | *p* value |
| --- | --- | --- | --- | --- |
|  | Age | 5.93 (1.14) | 5.22 (1.02) | 0.087 |
|  | EQ | 15.58 (7.0) | 13.33 (5.4) | 0.437 |
|  | CE | 4.42 (2.8) | 2.67 (3.0) | 0.149 |
|  | AE | 4.08 (2.4) | 2.50 (2.0) | 0.063 |
|  | SRS | 72.25 (21.4) | 87.50 (22.1) | 0.083 |
|  | CARS | 32.25 (2.4) | 36.17 (4.0) | 0.002** |
| CE condition | Within-CE network | 0.327 (0.17) | 0.396 (0.14) | 0.352 |
| AE condition | Across-networks | 0.238 (0.14) | 0.251 (0.15) | 0.628 |
| General conditions | Within-CE network | 0.324 (0.17) | 0.364 (0.16) | 0.659 |
|  | Across-networks | 0.241 (0.11) | 0.247 (0.13) | 0.796 |

*Note,* Means are reported with standard deviations in parentheses. ASC, autism spectrum conditions; ID, intellectual disability; EQ, Empathy Quotient; CE, cognitive empathy; AE, affective empathy; SRS, Social Responsiveness Scale; CARS, Childhood Autism Rating Scale.

** p<0.01.

**Table S3.** Correlation analysis between mean network functional connectivity strength and AE, CE, SRS and CARS scores both in ASC group and TD group

| Group | situation | Network connection | CE | AE | SRS | CARS |
| --- | --- | --- | --- | --- | --- | --- |
| ASC | CE situation | Within-CE | -0.144 | -0.177 | 0.081 | -0.082 |
|  | AE situation | across | -0.266 | -0.256 | -0.112 | 0.060 |
|  | General situation | Within-CE | -0.103 | -0.095 | -0.079 | -0.094 |
|  |  | across | -0.282 | -0.199 | -0.123 | -0.014 |
| TD | CE situation | Within-CE | 0.096 | 0.051 | -0.045 | - |
|  | AE situation | across | 0.307 | 0.272 | -0.257 | - |
|  | General situation | Within-CE | 0.238 | 0.201 | -0.134 | - |
|  |  | across | 0.068 | 0.087 | -0.004 | - |

*Note,* ASC, autism spectrum conditions; CE, cognitive empathy; AE, affective empathy; CE, cognitive empathy; AE, affective empathy; SRS, Social Responsiveness Scale; CARS, Childhood Autism Rating Scale.

**Sample Size Calculation**

To determine whether our study had sufficient statistical power to detect group differences, we performed a power analysis based on a previous fNIRS study reporting altered functional connectivity that was significant at the 0.05 level while watching a cartoon in autistic children (Li & Yu, 2016). Based on the information from this study (i.e., *df* = 22, critical *t*-value = 2.07), it could be inferred that the minimum effect size of group differences was Cohen’s *d* = 0.88. With an alpha level of 0.05 and power of 0.80, using G*Power (version 3.1.9.7) (Faul et al., 2009), the projected sample size needed with this effect size was estimated to be 22 individuals per group. Thus, our study was deemed to sufficient statistical power to detect differences in functional connectivity between groups.

**References**

Faul, F., Erdfelder, E., Buchner, A., & Lang, A.-G. (2009). Statistical power analyses using G*Power 3.1: Tests for correlation and regression analyses. *Behavior Research Methods, 41*(4), 1149-1160.

Li, Y., & Yu, D. (2016). Weak network efficiency in young children with Autism Spectrum Disorder: Evidence from a functional near-infrared spectroscopy study. *Brain and Cognition, 108*, 47-55.
